# Coping With Stress: The Mitokine GDF-15 as a Biomarker of COVID-19 Severity

**DOI:** 10.3389/fimmu.2022.820350

**Published:** 2022-02-16

**Authors:** Darakhshan Sohail Ahmed, Stéphane Isnard, Carolina Berini, John Lin, Jean-Pierre Routy, Léna Royston

**Affiliations:** ^1^ Infectious Disease and Immunity in Global Health Program, Research Institute of McGill University Health Centre, Montreal, QC, Canada; ^2^ Chronic Viral Illness Service, McGill University Health Centre, Montreal, QC, Canada; ^3^ CIHR Canadian HIV Trials Network, Vancouver, BC, Canada; ^4^ CONICET - Universidad de Buenos Aires, Instituto de Investigaciones Biomédicas en Retrovirus y SIDA (INBIRS), Buenos Aires, Argentina; ^5^ Division of Hematology, McGill University Health Centre, Montreal, QC, Canada; ^6^ Division of Infectious Diseases, Geneva University Hospitals, Geneva, Switzerland

**Keywords:** GDF-15, COVID-19, biomarker, disease tolerance, adaptive metabolic response

## Abstract

Growth differentiation factor 15 (GDF-15) is a transforming growth factor (TGF)-β superfamily cytokine that plays a central role in metabolism regulation. Produced in response to mitochondrial stress, tissue damage or hypoxia, this cytokine has emerged as one of the strongest predictors of disease severity during inflammatory conditions, cancers and infections. Reports suggest that GDF-15 plays a tissue protective role *via* sympathetic and metabolic adaptation in the context of mitochondrial damage, although the exact mechanisms involved remain uncertain. In this review, we discuss the emergence of GDF-15 as a distinctive marker of viral infection severity, especially in the context of COVID-19. We will critically review the role of GDF-15 as an inflammation-induced mediator of disease tolerance, through metabolic and immune reprogramming. Finally, we discuss potential mechanisms of GDF-15 elevation during COVID-19 cytokine storm and its limitations. Altogether, this cytokine seems to be involved in disease tolerance to viral infections including SARS-CoV-2, paving the way for novel therapeutic interventions.

## Introduction

Discovered in 1990, growth differentiation factor 15 (GDF-15) is a stress-induced cytokine and a distant member of the transforming growth factor β (TGF-β) superfamily (1–5). GDF-15 is the product of a gene on human chromosome 19p13.11-13.2 that was cloned in 1997 based on expression induction upon macrophage activation (6, 7). GDF-15 is considered as a major regulator of appetite (8–10) through its hindbrain receptor glial-derived neurotrophic factor receptor alpha-like (GFRAL), and its plasma levels were found to be elevated in the context of obesity and diabetes (11). However, GDF-15 acts more as a regulator than an inducer of obesity, as illustrated in mouse models where GDF-15 overexpression and administration of recombinant GDF-15 decrease glucose intolerance and enhance lipid metabolism (12). In addition to a metabolic function, GDF-15 recently emerged as an inflammation-induced mediator of disease tolerance through cellular metabolic reprogramming in the context of infections (13). Indeed, animal models showed that during acute infections, GDF-15 promotes survival by stimulating hepatic sympathetic outflow, which further promotes cardioprotective triglyceride production (13). Altogether, this cytokine seems to play a role in disease tolerance in bacterial and viral infections to a certain extent, including in SARS-CoV-2 infection, which opens up a new avenue for therapeutic intervention (13).

## The Cellular Production of GDF-15

GDF-15 was first named macrophage inhibitory cytokine-1 (MIC-1) since it was originally characterized in activated macrophages. Since, GDF-15 has been shown to be a cell-stress response cytokine produced in many cell types (7). Under physiological conditions, GDF-15 is expressed in high levels in the placenta, prostate, and bladder as well as other organs such as liver, kidney, lymph nodes, muscles and colon (14, 15). Cell types reported to express GDF-15 include adipocytes, cardiomyocytes, skeletal and smooth muscle cells and macrophages (15). L’homme et al. recently identified saturated fatty acids (SFAs) as strong inducers of GDF-15 expression in macrophages (11). Endoplasmic reticulum (ER) stress was identified as a key trigger of SFAs-induced GDF-15 expression, through the unfolded protein response (UPR) at the cellular level *via* PKR-like ER kinase (PERK) (11). Such findings suggest a link between GDF-15 and obesity, as SFAs-activated macrophages produce pro-inflammatory cytokines such as TNF-α and IL-1β (16). Additionally, GDF-15 was reported to be overexpressed in cancer cells of various origins, including predominantly prostatic, renal, urothelial, colorectal cancers and melanoma (17). Globally, GDF-15 is expressed in many different cell types from various organs, both in physiological and pathological conditions.

## Conditions Inducing Increased Circulating Levels of GDF-15

High circulating levels of GDF-15 have been associated with chronic inflammatory conditions including renal, lung, liver and cardiovascular diseases (4–11), rheumatoid arthritis, cancers, anemia (18) and infections such as COVID-19. Under physiological conditions, elevated plasma levels of GDF-15 are also reported in older individuals, in late pregnancy and during strenuous exercise. The use of certain drugs such as metformin and colchicine has also been independently associated with increased levels of GDF-15 (12, 19, 20). Furthermore, GDF-15 appears as a marker for all-cause mortality in the elderly and constitutes a predictor of disease severity during bacterial and viral infections (21, 22). During inflammatory conditions, multiple cell types have been shown to release GDF-15, including endothelial cells, epithelial cells, vascular smooth muscle, macrophages and adipocytes (4, 23, 24). Taken together, GDF-15 appears to be released by multiple cell types both during acute and chronic low-grade inflammation.

## GDF-15 as a Mitokine During Mitochondrial Dysfunction

Mitochondria are intracellular organelles that constitute cellular “power stations” in all cell types and tissues. These organelles play key roles in many biological processes, such as programmed cell death, oxidative phosphorylation and energy production. Aging and inflammation have been shown to alter mitochondrial function (3, 25).

Upon stress, mitochondrial stress-induced cytokines (referred as mitokines) such as fibroblast growth factor 21 (FGF21) and GDF-15 are expressed (26). Mitokines act in an endocrine, paracrine and autocrine fashion depending on the tissue microenvironment, and have been shown to have both detrimental and protective effects depending on the stimulus intensity (11, 21). GDF-15 is involved in a biphasic (hermetic or U-curve) response *via* the GDF-15-STAT3 pathway (2, 21, 27). Such hormetic paradoxical dose-response has been illustrated by Conte et al. who demonstrated that low plasma levels of GDF-15 were associated with healthy ageing, while higher levels were detrimental (2, 21). GDF-15 production has been associated with a low NAD/NADH ratio, highlighting the influence of mitochondrial dysfunction on GDF-15 production (28), although ER stress and hypoxia were also shown to induce this cytokine (2, 29).

## Cellular Mechanisms and Downstream Signaling Pathways of GDF-15

There are still conflicting reports of the molecular mechanisms of GDF-15 at the cellular level (2, 30, 31). It is established that GDF-15 binds to the GDNF receptor family member GFRAL in the hindbrain, orchestrating its metabolic effects through the neurotransmitter cholecystokinin (CCK) and further reduces appetite and body weight through the RET coreceptor (8, 10, 31, 32). However, GFRAL is not expressed outside the brain, raising the possibility of alternative receptors involved in GDF-15’s immunomodulatory effects, especially through hematopoietic cells (31). As a member of the TGFβ family, it has been speculated that the peripheral effects of GDF-15 could be mediated through receptors of the TGF-β/Smad signaling pathway (2, 31, 33, 34). Candidate receptors for GDF-15 are ALK-5/TGF-βRII, TGF-βRI, TGF-βRII and the epithelial growth factor receptor ErbB2 (35–37). Recently, a new receptor for GDF-15, CD48, has been identified on Treg cells in a genetically-engineered mouse model (38). The GDF-15-CD48 interaction was shown to promote the propagation of Treg cells and indirectly upregulate forkhead transcription factor (Foxp3), enhancing the development of hepatocellular carcinoma (HCC) (38). This immunosuppressive tumor microenvironment was further shown to be altered by the introduction of monoclonal antibodies against GDF-15, which ultimately improved HCC control (38).

Downstream signaling of GDF-15 has been studied in different models. In a mouse model, GDF-15 has been shown to activate the PPARβ/δ-AMPK-p53 pathway, enhancing the fatty acid oxidation and glucose uptake and reducing ER stress as well as inflammation (39). Interestingly, the antidiabetic effect of PPARβ/δ was independent of the central GDF-15/GFRAL receptor in the hindbrain (39). Moreover, GDF-15 was reported to contribute to the increase in peroxisome proliferator-activated receptor-gamma coactivator (PGC)-1α and lipin-1, involved in fatty acid metabolism induced by PPARβ/δ activation (39). In the same animal model, the expression of GDF-15 was induced in the skeletal muscles *via* PPARβ/δ agonist, mitigating inflammation and improving glucose tolerance (39). In another mouse model, GDF-15 induction through the PPARγ pathway plays a key role in tissue regeneration (40). In summary, hindbrain GFRAL acts as a receptor for GDF-15 and may explain part of its metabolic effects, however the mechanisms of its immunomodulatory effect remain to be determined.

## Coping With Stress: GDF-15 in the Context of Host Resistance and Disease Tolerance

Studies on GDF-15 levels during sepsis illustrate the hormetic role of GDF-15 during infection, with low levels being protective while high levels being associated with disease severity. Host survival during infection requires a delicate balance between host resistance, which is essential for detecting and eliminating pathogens, and disease tolerance, which is critical in minimizing collateral tissue damage (41). During infection, mortality is mainly determined by an exaggerated immune response rather than pathogen invasion, reflecting this dysregulation of the balance between defense and tolerance (42). Disease tolerance can be then perceived as “the beauty of compromise” as Mahatma Gandhi stated in his autobiography written by Louis Fisher in 1950, illustrated by T-cell exhaustion and metabolism reprogramming (Warburg effect) in cancer and chronic infections. GDF-15, which has emerged as an inflammation-induced mediator of disease tolerance through metabolic reprogramming, might then serve as a disease tolerance cytokine. The balance between disease tolerance and host-defense response is particularly relevant for COVID-19, due to the negative impact of a hyperinflammatory state in COVID-19. This phenomenon is best illustrated in bats, which are well-documented viral reservoirs and harbor many zoonotic coronaviruses. Despite high viral loads of highly pathogenic viruses in humans (as Ebola virus or SARS-CoV), infected bats exhibit no signs of disease (43, 44).Through unique immune characteristics, bats have an excellent balance between host defense and disease tolerance, allowing them to tamp excessive immune responses to pathogens (45) Effector molecules with known immunomodulatory effects such as GDF-15 might then be of great interest to restore a balanced response.

## Association Between Higher GDF-15 Plasma Levels and Respiratory Tract Diseases

Pulmonary epithelial cells constitute a major source of GDF-15 production (46, 47), especially during hypoxia (48) or upon exposure to various allergens, cigarette smoke (49) and air pollutants (50). In chronic obstructive pulmonary disease (COPD), which is associated with cigarette smoking, a positive association has been found between elevated levels of GDF-15 and exacerbation frequency as well as impairment of pulmonary function (7, 51–53). During pulmonary hypertension, GDF-15 has been associated with disease progression and mortality. Higher levels of GDF-15 have been linked with increased atrial pressure and pulmonary capillary wedge pressure, *via* induction by hypoxia and shear stress from the pulmonary vascular endothelial cells (46, 54). In addition, higher GDF-15 plasma levels were also found in alveolar epithelial cells in pulmonary fibrosis (47, 55). Tissue damage due to hyperoxia is also a strong inducer of GDF-15 secretion by pulmonary epithelial and endothelial cells and is linked with bronchopulmonary dysplasia (56). Conversely, GDF-15 demonstrated a protective role in ventilator-associated acute lung injury induced by platelets-neutrophils aggregates (48).

## The Significance of GDF-15 in Viral and Bacterial Infections Outside COVID-19

GDF-15 levels are significantly increased in patients with various infections and sepsis. Regarding hepatic viral infections, GDF-15 is associated with disease progression and enhanced viral replication (57–60). Hepatitis B virus (HBV)-linked HCC (57, 59–61) and liver fibrosis linked with hepatitis C virus (HCV) were both associated with GDF-15 elevation (58, 60, 62). GDF-15 was reported to be overexpressed in genetically engineered mice with acute exacerbations of COPD due to human rhinovirus (RV), the most frequently detected virus in this context (63, 64). Moreover, in human airway epithelial cells as well as in a mouse model, GDF-15 was shown to promote RV replication and to increase viral-induced inflammation (64). This increased inflammation, which is known to be related to symptoms, could be partly explained through the impairment of interferon-γ1 (IFN-γ1) production by GDF-15 (64).

Although less studied, bacterial infections have also been associated with increased GDF-15 levels, both for Gram-positive or Gram-negative bacteria (13, 65) Similarly, GDF-15 was found to be elevated in patients with septic shock and its plasma levels were correlated with increased mortality (66). On the other hand, GDF-15 knockout mice were shown to be protected against severe septic infection, with prolonged survival, and demonstrated better control over local infections (61, 66).

## GDF-15 as a Biomarker Coming of Age in the COVID-19 Pandemic

Several proteins have been identified as prognostic biomarkers in COVID-19 such as IL-6, IL-8, C-reactive protein (CRP), procalcitonin, ferritin, D-dimer, calprotectin, IFN-γ-induced protein 10 (IP-10), IFN-γ, TNF-α, granulocyte monocyte-colony stimulating factor (GM-CSF), and macrophage inflammatory protein (MIP) 1α and 1β (66–71). However, only a handful of these cytokines were coined as prognostic markers associated with disease severity and progression in COVID-19 patients (reviewed in **Table 1**) (74). In addition, due to their very short half-lives, accurate cytokine measurements in the plasma remain difficult and need to be carefully interpreted.

**Table 1:** Studies reporting an association between GDF-15 and COVID-19 severity.

Study design and country	Demographics	Comorbidities of studied patients	Sample size (n)	Patients with severe Sx/MR	Plasma GDF-15 correlation with patient status	Other markers correlated withn GDF-15	Authors
Observational study	Hospitalized patients	DM, HTN, CVD, CKD, non-asthma respiratory disease, immunosuppression	66	8/12.1%	Severity of the disease	Calprotectin	de Gaudiana et al. (68)
Spain	> 60 years				Mortality		
	Mostly males						
Prospective observational study	ICU-hospitalized patients	DM, HTN, CVD, CKD, COPD, obesity	123	35/28%	Severity of the disease at baseline, day 3, day 9	Ferritin	Myhre et al. (70)
Norway	>18 years				ICU admission		
					Mortality		
Single-center retrospective study	Presence of ARDS	None	39	15/38.8%	ICU admission	IL-6, IL-10 and CRP	Notz et al. (71)
Germany	Median age 58 years						
	77% males						
Case-control study	Hospitalized patients (moderate-severe symptoms) Median age 52 -58 years	None	80 (patients with varying disease severity and Control)	20/10%	Hospitalization rate	C3a, galectin-9	Giron et al. (69)
USA	50% females				Mortality		
Cohort study	Median age 71 years	CVD, DM, HTN, stroke, prior MI, current smoker, obese	3999 and 1088 (2 different international cohorts-ARISTOTLE and RE-LY studies)	ND	Mortality risk	NT-proBNP	Wallentin et al. (72)
Sweden, USA	73% males						
Cohort study and subcohort study	Median age 72.2 years in ESKD and COVID-19 + group		Subcohort A - - 55 COVID-19 positive ESKD patients	41/46, 89%	Disease severity	IL18BP, CTSD and KRT19	Gisby et al. (73)
London, UK	Median age 70.1 years in ESKD and COVID-19 - group		- 51 COVID-19 negative ESKD patients		Mortality risk		
			Subcohort B				
			- 52 COVID-19 positive ESKD patients				
			- 11 COVID-19 negative ESKD patients				
Retrospective study	Median age 38-62 years	HTN, DM, anemia, liver cysts, respiratory diseases, stroke, CVD, hyperlipidemia	440	Males= 10 (56%)	Severity and progression of disease	IL-6, IL-8 and CRP	Teng et al. (6)
Foshan, China			(biomarkers analyzed in 111 patients)	Females= 21 (64%)			

GDF-15, growth differentiation factor 15; HIV, human immunodeficiency virus; HBV, hepatitis B virus; HRV, human rhinovirus; IL, interleukin; CRP, C-reactive protein; GM-CSF, granulocyte-macrophage colony-stimulating factor; TNF, tumor necrosis factor; IFN, interferon; DM, diabetes mellitus; CKD, chronic kidney disease; HTN, hypertension; MI, myocardial infarction; COPD, chronic obstructive pulmonary disease; CVD, cardiovascular disease; O2, oxygen; CLD, chronic liver disease; C3a, complement 3a; HCC, hepatocellular carcinoma; HBV, hepatitis B virus; CLD, chronic liver disease; CHB, chronic hepatitis B; ESKD, end-stage kidney disease; ARDS, adult respiratory distress syndrome; MR, mortality rate; (+), high plasma levels; hs-cTnT, high sensitive cardiac troponin T; NT-proBNP, N-terminal proB-type natriuretic peptide; n, sample size; UK, United Kingdom; CTSD, cathepsin D; KRT19, keratin 19; ICU, intensive care unit; O2, oxygen; Sx, symptoms; MR, mortality rate; ND, not described.

Through the correlation of tissue damage, hypoxia and aging, GDF-15 emerged as a significant indicator of disease severity in individuals infected with SARS-CoV-2 (**Table 1**) (6, 71, 73, 75), specifically in patients with underlying lung pathologies such as COPD in older individuals (42, 46). An inverse correlation has been reported between GDF-15 plasma concentration and oxygen saturation, leading to stratification of disease severity in critically-ill patients with COVID 19 (70). Notz et al. demonstrated that both IL-6 and CRP were correlated with GDF-15 levels throughout the COVID-19 course, suggesting the significance of GDF-15 in inflammation (71). Among many inflammatory markers, increased levels of GDF-15 and ferritin were associated with poor outcomes in the intensive care unit (ICU) and hospitalized patients with COVID-19 (70). Similarly, de Guadiana et al. demonstrated a positive correlation between GDF-15 and ferritin, CRP, calprotectin, and D-dimer in hospitalized COVID-19 patients (68). GDF-15 and calprotectin were found to be the best prognostic markers in assessing the outcome in hospitalized patients infected with SARS-CoV-2 (68, 70). Taken together, higher plasma levels of GDF-15, cardiac biomarkers and higher levels of soluble angiotensin-converting enzyme 2 (sACE2) have been proposed for risk stratification in patients with COVID-19 (67, 72). In patients with end-stage kidney disease (ESKD) infected with SARS-CoV-2, Gisby et al. found more than 200 proteins differentially expressed compared to non-infected controls, 67 of which were linked to an increased risk of mortality (73). Among various proteins known as contributors of inflammation and organ damage, GDF-15 was one of the top 12 cytokines/chemokines on the list (73).

In another study, out of 440 potential biomarkers tested by antibody array profile and confirmed by enzyme-linked immunosorbent assay (ELISA), GDF-15 was found to be consistently and statistically correlated with the severity and the progression of COVID-19 (6). Dynamic changes of GDF-15 levels reflected disease progression, with high levels linked to symptom deterioration, followed by a dramatic decline in plasma GDF-15 levels at the time of clinical and radiological improvement and discharge (6). This study indicates that GDF-15 could be used as a predictor of the progression of the disease (**Table 1**) (6).

## Immunomodulatory Function of GDF-15 During COVID-19

It has been established that the pathogenesis of severe COVID-19 involves the hyperactivation of the immune response leading to a life-threatening ‘cytokine storm’ (76). This clinical syndrome can be induced by both infectious and non-infectious causes, and is characterized by an imbalance between cytokine production and activation of the immune response leading to a hyperinflammatory state and multiorgan failure. Extensive cytokine surges, such as IL-1, IL-6, IL-18, TNFα and IFN-γ (76) triggered by various pathogens (77–79), induce a cytokine release syndrome (CRS) leading to widespread inflammation and considerable tissue damage (76, 80). This further leads to endothelial cell dysfunction, multiorgan failure, disseminated intravascular coagulation (DIC), acute respiratory distress syndrome and alteration in iron homeostasis (74, 76, 81). COVID-19 mortality is directly correlated with the elevation of cytokines (82), in which monocyte/macrophages, neutrophils, and natural killer (NK) cells seem to play a role (74, 76). Innate and adaptive immune responses have also been shown to be uncontrolled, specifically in virus-infected cells (76). In the case of COVID-19, a small proportion of patients affected by severe disease were shown to present with underlying dysregulated host innate response, inducing a hyperinflammatory syndrome (83). In addition, a cytokine storm is observed more frequently in elderly patients and correlates with rapid deterioration during COVID-19 (75, 84). Severe outcomes have been particularly observed in patients with coexisting chronic inflammatory conditions, such as hypertension, diabetes, and obesity, which are in turn linked with elevated plasma GDF-15 levels (76). Cytokine storm-induced GDF-15 elevation was shown to protect against cardiovascular alterations in a mouse model, however, it remains unknown whether this effect is present in COVID-19 patients (13).

**Figure 1:**
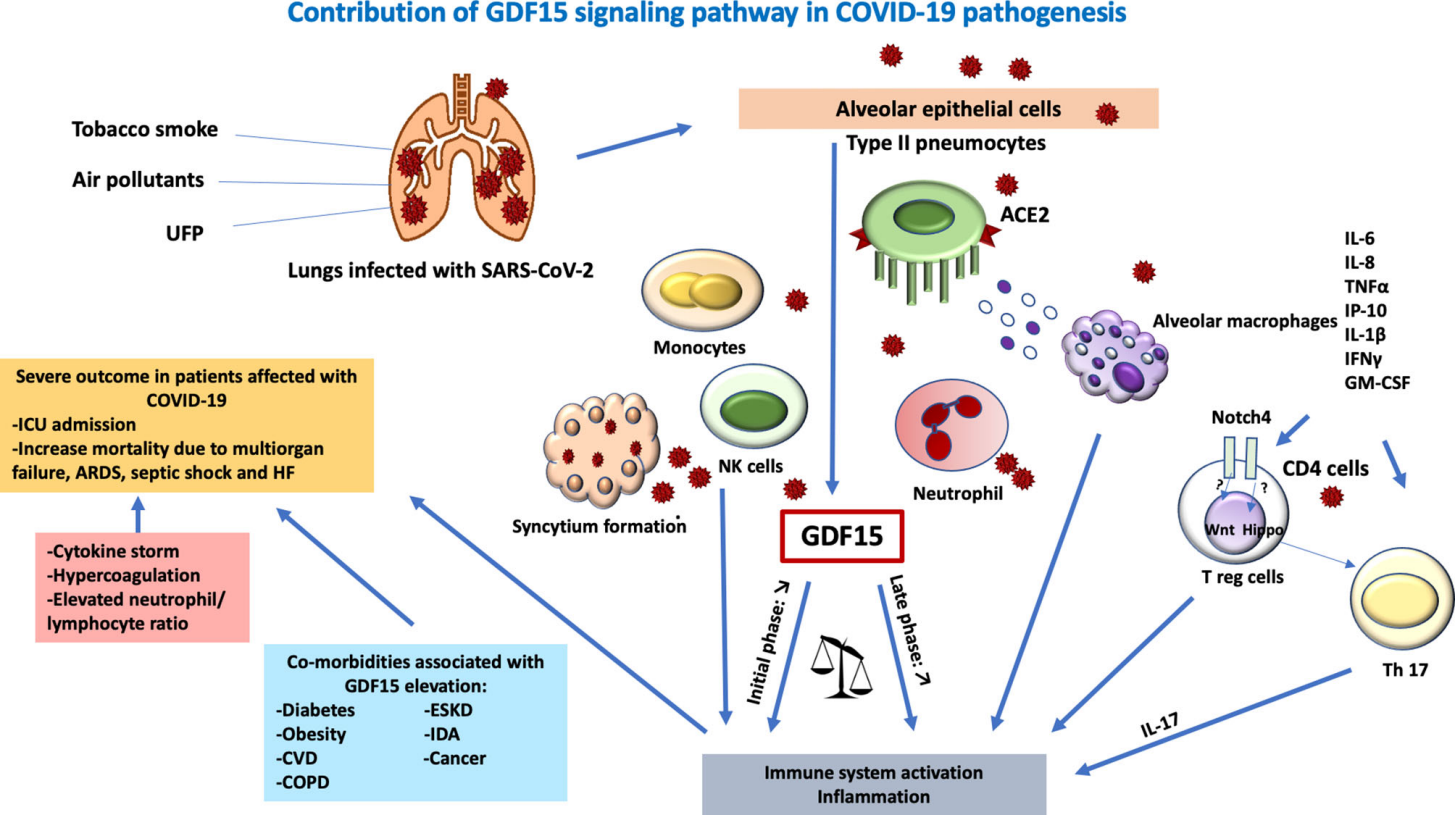
Contribution of a GDF-15 signaling pathway in COVID-19 pathogenesis. Lungs infected with SARS-CoV-2 lead to tissue damage, hypoxia, and endothelialitis. Tobacco smoke, ultrafine particles, and air pollutants act as a co-stimulant in the direct release of GDF15 in the lung epithelial cells. The virus enters the host cell *via* ACE2 on type II pneumocytes causing the recruitment of leucocytes, hence, elevated innate immune response. SARS-COV-2 also causes direct endothelialitis after the destruction of the alveolar epithelia. The transmigration of leucocytes causes a massive release of proinflammatory cytokines IL-6, IL-8, TNF alpha, IP-10, IL-1beta, IFN gamma, GM-CSF, and Notch pathway. The hippo pathway favors IL-17 differentiation and the Wnt pathway inhibits Treg suppressor function mediated by GDF15 resulting in overwhelming immune system activation. Together with the formation of the syncytium, hyperactivation of immune response commenced leading to cytokine storm, hypercoagulation and elevated neutrophils-lymphocytes ratio critical in severe outcomes in patients affected with SARS-COV-2 especially with comorbidities. GDF, growth differentiation factor; IL, interleukin; ARDS, acute respiratory distress syndrome; ACE, angiotensin-converting enzyme; UFP, ultrafine particles; HF, heart failure; COPD, chronic obstructive pulmonary disease; CVD, cardiovascular diseases; CD, cluster differentiation; GM-CSF, granulocyte monocyte-colony stimulating factor; SARS-CoV-2, severe acute respiratory syndrome coronavirus 2; ESKD, end-stage kidney disease; IDA, iron-deficiency anemia; T reg, regulatory T cells; ICU, intensive care unit; NK cells, natural killer cells.

Endothelial dysfunction is also a hallmark of COVID-19 and has been linked with oxidative stress (74, 85). The hyperactivity of the angiotensin-converting enzyme (ACE)-Angiotensin (Ang) II- Angiotensin type 1 receptor (AT1) axis of the classical renin–Ang system was shown to contribute to the coagulopathy observed in patients with COVID-19 (74). In addition, endothelial cells constitute a direct target of SARS-CoV-2, which further contributes to endothelial dysfunction. The SARS-CoV-2 cellular receptor ACE2 is heavily expressed in vital organs such as the lung, liver, kidneys, heart, and blood vessels, especially in type II pneumocytes in the lungs (74). Upon binding, the virus is internalized through the endogenous ACE2 receptor through the S1 domain of the spike glycoprotein (S) (74). S2 domains expressed on the SARS-CoV-2-infected cells then cause a fusion between ACE2-positive neighboring cells and triggers the formation of multinucleated syncytial pneumocytes (74, 86). AT1 receptor plays a pivotal role in oxidative stress through numerous intracellular signaling pathways. The endothelial cell damage causes recruitment of inflammatory cells and overproduction of cytokines and endothelialitis resulting in microcirculatory vascular changes in the various tissues (74).. Due to its high expression in endothelial cells and its induction upon hypoxia, GDF-15 might play a role in COVID-19 endothelialitis (46, 54, 70). GDF-15 is secreted from the epithelial and endothelial cells as a result of inflammation and oxidative stress in COVID-19. GDF-15 may exert its effect directly on immune cells as well as *via* the central GDF-15/GFRAL receptor in the hindbrain (74). The high amount of IL-6 secreted by activated macrophages trigger production of IL-17, which results in excessive immune activation and intense widespread inflammation (50) (**Figure 1**). Despite its involvement in immune tolerance, GDF-15 elevation seems to be overwhelmed by uncontrolled inflammation in certain patients with COVID-19, leading to vascular pathologies in vital organs (74, 87, 88).

## GDF-15, Iron Metabolism and COVID 19

One of the proposed mechanisms of oxidative stress and hyperinflammatory state in COVID-19 is dysregulation of iron metabolism (74). Plasma GDF-15 levels have been found to be high in iron deficiency anemia, anemia of chronic disease and iron overloading anemia such as β-thalassemia (89–91). GDF-15 is inversely associated with hepcidin, a key regulator in systemic iron homeostasis in mammals (74, 90, 92), expediting intestinal iron absorption leading to iron overload (93, 94). Inflammation, which is also a hallmark of chronic anemia, increases the expression of GDF-15 in several pathologies such as ESKD, cancers, diabetes and cardiovascular diseases (73, 91, 94, 95) (**Table 1**). GDF-15 has emerged as an immune modulator in older patients with anemia in COVID-19 and its role is critical in ferroptosis and dysregulated hematopoiesis in the erythroid cell lineage (74, 96).

Iron deficiency anemia is very common in patients with ESKD (97). In one cross-sectional study in South Africa, GDF-15 was found to be a predictor of iron deficiency anemia in early renal disease (91). GDF-15 is also associated with a decline in renal function in chronic renal diseases (98). The iron overload associated with overexpression of GDF-15 in inflammatory states could lead to increase ferritin, another crucial biomarker in stratifying disease severity in COVID-19 (**Table 1**). Altogether, this could partly explain the relationship between the elevated plasma GDF-15 levels, underlying anemia, and severity of COVID-19 in chronic inflammatory conditions especially ESKD (73, 74).

Recently, high-dose iron chelation has been approved by FDA as adjuvant therapy in critically-ill patients infected with SARS-COV-2 (18). In addition to lowering iron levels, iron chelating therapy demonstrated antiviral and antifibrotic activity while improving endothelialitis and innate immunity (99). There is some evidence supporting treatment of COVID-19-associated-mucormycosis with iron chelators (100), although more studies are needed to fully understand the beneficial effects of this adjuvant therapy.

GDF-15 could therefore potentially be used as a critical biomarker to predict the early use of iron-chelating therapy in patients with COVID-19 with co-existing subclinical inflammation and complications.

## Conclusion

Altogether, high levels of GDF-15, a stress-related cytokine, have been associated with the progression and severity of various conditions including COVID-19. Based on our literature review, GDF-15 represents a clinically relevant marker for risk stratification or screening for severe COVID-19 (41). The use of GDF-15 as a biomarker could also enable the identification and optimal timing of the most appropriate therapies in patients with COVID-19 (87). The role of GDF-15 in viral pathogenesis, notably COVID-19, seems to be context-dependent, spanning from a promotor of disease tolerance in the early phase of infection to a detrimental actor in certain patients with cytokine storm. Furthermore, the potential outcome of treating early COVID-19 patients with recombinant GDF-15 could be explored in further studies.

## Author Contributions

DA contributed to the planning of the manuscript, reviewed the literature and wrote the first draft of the manuscript. SI, JL and CB reviewed the first draft and approved the final draft of the manuscript. J-PR and LR contributed equally, conceived and designed the manuscript, contributed to the literature review, reviewed the manuscript drafts, and approved the final version. DA, SI, JL, CB, LR, and J-PR provided critical revision of the manuscript. All authors approved it for publication.

## Funding

Our research is funded by the Fonds de la Recherche Québec-Santé (FRQ-S): Réseau SIDA/Maladies infectieuses and Thérapie cellulaire; the Canadian Institutes of Health Research (CIHR; grants MOP 103230 and PTJ 166049); the Vaccines & Immunotherapies Core of the CIHR Canadian HIV Trials Network (CTN; CTN PT038); CIHR- funded Canadian HIV Cure Enterprise (CanCURE) Team Grant HB2-164064. LR is supported by a Postdoc mobility grant of the Swiss National Science Foundation, Switzerland and a research fellowship from the Canadian Institutes of Health Research (CIHR) HIV trial network (CTN). SI is supported by research fellowships from Fonds de la Recherche Quebec-Science (FRQ-S) and the CIHR/CTN. J-PR is the holder of the Louis Lowenstein Chair in Hematology and Oncology, McGill University.

## Conflict of Interest

The authors declare that the research was conducted in the absence of any commercial or financial relationships that could be construed as a potential conflict of interest.

## Publisher’s Note

All claims expressed in this article are solely those of the authors and do not necessarily represent those of their affiliated organizations, or those of the publisher, the editors and the reviewers. Any product that may be evaluated in this article, or claim that may be made by its manufacturer, is not guaranteed or endorsed by the publisher.
